# Chicago Medical Society; Stated Meeting of April 18th

**Published:** 1887-06

**Authors:** 


CHICAGO MEDICAL SOCIETY.

*Stated Meeting, April 18th, 1887.*—THE PRESIDENT, W. T. BELFIELD, M. D., in the chair.

DR. FRANCIS J. CRANE read the following paper entitled

### BERGEON'S METHOD OF TREATING PHTHISIS BY GASEOUS ENEMATA.

In January last the *New York Medical Record* contained an article headed "A New Treatment of Phthisis," which gives a brief outline of Dr. Bergeon's method of administering carbon dioxide mixed with sulphuretted hydrogen gas, and referred to an article published in the *British Medical Journal* of December 18, 1886. This described in full the apparatus, and mode of using it, and stated they had been procuring beneficial results by the method, and that Professor Cornil, of Paris, had also become an enthusiastic supporter of it. This led me to write to Dr. Bergeon, and in the course of our correspondence he presented me with an apparatus as well as with the treatise of Dr. Morel read before the French Academy last June, and also one by Professor Cornil. These are so exhaustive that I have embodied in this paper only the essence of both, as follows: "New Treatment of the Diseases of Respiratory Organs and Septicœmia by the Means of Gaseous Enemata according to M. Bergeon's Method," by Dr. V. Morel. It would seem from the statements of physicians who have tried either to prevent the development, or the proliferation of the bacillus, or to destroy it, that it is the one thing which we have to overcome in the treatment of phthisis. In reasoning by analogy with other contagious diseases—as, for instance, cholera and hydrophobia—it might be urged that the bacillus is not the immediate cause of the morbid phenomena of tuberculous affections. It is known that, aside from miliary tubercles, which invade the lungs and are the cause of the patients' symptoms, the gravest phenomena of phthisis are due to the septicæmia, which poisons the patient, and is caused by the suppuration of the tubercles, which, brought in contact with the air, undergoes putrefaction, and is absorbed into the system. The bacillus work, then, by producing lesions of texture, which become fatal to the organism by rapidly destroying, or by undergoing softening and absorption, produce septicæmia. To use Darembourg's expression: "The bacillus is nothing, but the septicæmia is everything."

In acknowledging that it is not necessary to ascribe to the microbe all of the morbid phenomena of phthisis, it is not less true that its presence is to the organism an incessant and real danger, and that consequently, in endeavoring to discover a remedy for the lesions which it has produced, it is necessary to destroy it, or at least to neutralize its action. For this purpose such agents as sulphuret of hydrogen, bisulphide of carbon and other antiseptics mixed with pure carbonic acid, are employed.

Principles of Bergeon's Method.—The first mode of treatment thought of consisted in applying by inhalation some substances having parasiticidal properties. It is known that antiseptic substances are poisonous when introduced into the arterial system, either directly or by inhalation. Claude Bernard has shown that poisonous gases introduced into the arterial system through the lungs produce toxic effects almost instantaneously Besides, the antiseptic substances have an irritating action, and this action,. operating on diseased lungs, will only increase the existing lesions, while their unpleasant odor arouses a refusal in the patients to their use; this is probably the reason why so little success has been obtained by inhalation in the treatment of phthisis, and is conclusive proof that the introduction of antiseptics by the stomach is preferable, for Claude Bernard has shown that when a poisonous or mineral substance is introduced into an organ distant from the arterial system—into the digestive tract, for instance—it cannot enter the arterial system, because it is expelled before reaching it. It must pass through the portal system, the hepatic veins and the pulmonary texture, there to be exhaled, or it can be expelled in the liver with the bile.

It is well established by experiments that the introduction of poisonous substances into the digestive tube may be done without danger by taking certain precautions, of which the most important consist in not injecting too large quantities at once, and not injecting more before the first has been completely eliminated. What avenue ought to be chosen? The stomach or the rectum? In both cases the medicament will have to pass through the portal vein, the liver, the hepatic vein, the right heart and pulmonary arteries, but we think the rectal way is preferable, for the patient cannot take a dislike to the antiseptic substances on account of their disagreeable odors.

"Gaseous Enemata in the Therapeutics of Respiratory Organs, Pulmonary Phthisis, Asthma, Whooping-cough, Bronchitis, Bronchiectasis, Bronchorrhæa, Pulmonary Catarrh," by M. Cornil. The principle of the action of gaseous infections and of their rapid elimination by the lung has been given by Bernard. He showed that when sulphuretted hydrogen is injected into the rectum of an animal, the gas is expelled by the lungs; he proved that we can so inject it in almost umlimited quantities without causing harm; whereas its introduction by inhalation rapidly occasions grave accidents and the animal's death. However, to introduce sulphureous hydrogen, or gas or any other vapor, into the economy per rectum, for the purpose of destroying the micro-organisms which exist in a number of diseases, it was necessary to find a gaseous vehicle inoffensive to the economy and easily tolerated by the bowel.

Carbonic acid gas admirably answers the purpose; it is very easily borne by the colon, rapidly absorbed, and afterward expelled by the lung, with the medicinal gas which it holds. The gas itself, in all probability, plays a very important part in this new treatment of pulmonary diseases.

Dr. Bergeon, who inaugurated this method, published a few months ago the first results obtained in the treatment of pulmonary phthisis by this method. Physicians of Lyons, Paris, Geneva and Marseilles who have treated phthisis by the method have generally obtained a very rapid disappearance of the phenomena of pulmonary suppuration, and a progress towards a state of health, with all the signs of cure.

Concerning the patients I have treated by this method, I can now assert that the results I predicted three months ago have been achieved. The patients that I considered cured have no more expectoration, and give on auscultation stethoscopic signs which denote the presence of quiescent cavities or cicatrized lesions. Some of these patients have been obliged to return to a life of labor; nevertheless their respiratory organs have stood the test, and the amelioration obtained has been permanent.

While many patients whom the expectoration once so exhausted now have only 3 or 4 grams of sputum a day, at the beginning of the treatment it was from 250 to 300 grams. We have found bacilli, it is true, in the sputa of these patients; yet it remains to be discovered whether these bacilli which exist after the return to health have kept their functional activity or not. Whatever may be the mode of action of carbonic acid introduced by intestinal absorption in, the venous blood and afterward expelled by the lung, it can be said from the observation of patients that this gas, filled with proper medicinal substances, greatly modifies the respiratory functions, and makes the hemathosis more complete and easy. It gives a sensation of well-being, followed by an increase of strength and weight, a diminution of fever and night sweats.

The following precautions must be observed in giving this treatment:

1. The $CO^2$ ought to be as pure as possible, so as not to inflame the bowel. That obtained by the reaction of dilute sulphurlc acid on the bicarbonate of soda has always been perfectly absorbed by the bowel without producing any toxic effect.

2. The gas should be collected in a receiver from which the air has been expelled.

3. Make the injections just before a meal, or at least three hours after, and never when the patient is weary. It is necessary to be very cautious in experimenting with other medicinal substances, for if, although the sulphuretted hydrogen is inoffensive, other agents, as turpentine, chloral, ammoniæ, iodine, bromæ, ether, etc., may not be, and might be the cause of an inflammation of the intestinal mucous membrane.

It is not necessary that the dose be large; by injecting twice a day 4 or 5 litres of carbonic acid gas passed through 500 grams of sulphur water, we rapidly notice the disappearance of all the phenomena of pulmonary suppuration, either in its acute or chronic state.

Bergeon's method has been successfully experimented with by Dr. Chantamesse, in his service at St. Antoine Hospital, during the months of August, September and October. The following are his results: "Two patients brought to the hospital suffering with violent attacks of asthma were, half an hour after the injection with sulfo-carbon vapor, entirely relieved of the dyspnœa. The treatment having been continued for a few days, the breathing was relieved, and the attacks were not repeated during the time they remained." Nine patients giving general and local signs of pulmonary tuberculosis, with tubercular bacilli in the sputa, have obtained very great amelioration from this treatment. The increase of weight has been rapid, one pound, and sometimes as high as 35 oz. a week; cough and expectoration have largely ceased. We also find the bacilli in the sputum however. These patients have been under treatment for one month and a half. One of them has increased nine pounds in weight.

I have used this treatment with four cases; two of phthisis, one of intussusception of the bowel and one of spasmodic croup. With the croup and intussusception it operated like a charm, overcoming both almost instantly. In the case of croup, I used the bisulphide of carbon, and in half an hour the little patient was sleeping, apparently as well as ever.

Case 1.—Mr. W., aged 26. Two sisters and a brother died of phthisis; he had been treating with various physicians and changing climate (having been to Colorado twice) for over three years. The right lung was nearly useless, as it contained a cavity corresponding to nearly if not quite half of its original capacity. Nowhere on that side could vesicular respiration be heard, while the left apex likewise yielded unmistakable signs of disease. There were œdema of the feet, incessant cough, broken sleep, watery stools and ravenous appetite, although he could not retain anything on the stomach; temperature 102° F. After the first injection of bisculphide of carbon, given in the evening, he slept well for three hours and was bothered very little with cough, but on rising in the morning, to use his own words, came nearly strangling for want of a cough, which be finally got, and expectorated a pint with the one paroxysm. I then used the sulphuretted hydrogen water and he improved very fast; in one week he had a normal temperature; night sweats almost entirely stopped, expectoration was much less, and he was able to wear his shoes, which he had not been able to do for over six weeks. Unfortunately, however, at the latter part of the second week he ventured out in one of our rainiest March days, took cold, and his death, two days later, cut short the record of what might have proven almost a miracle.

Case 3.—Mrs. W., aged 34, widow, having lost her mother and oldest sister from phthisis, applied to me for some heart trouble. Complained of a dizzy sensation on rising from a recumbent position; feet swollen some, hectic flush, considerable dysphnœa, slight cough with no expectoration. *Diagnosis:* Incipient phthisis with heart complication? She had noticed, also, for about a week some night sweats, which did not last, however, after the second administration of gas. She improved so rapidly that she only made seven visits in all, and pronounced herself cured. There is, however, no doubt but she will have a return of symptoms upon the slightest provocation.

This comprises all my experience, but these are facts, and facts are stubborn things to deal with. In regard to the best mineral water, I wish to say that, after trying the Lafayette, Ind., the Blue Lick, Ky., and the Ypsilanti, Michigan, mineral waters, I am satisfied that the Ypsilanti mineral water is just what we want. It contains 20 cubic inches of gas to the gallon, and is so strongly impregnated with it that I use it over the second time by having solid rubber corks to replace the perforated ones when I have got through using the apparatus. Mr. St. Clair, President of the Company has kindly furnished a case of twelve quarts of the water, which I have forwarded to Dr. Bergeon, in hopes that it will compare favorably with the Eaux Bonnes water which he is using. I have also had an apparatus made by E. H. Sargent which I think takes the place of Dr. Morel's very well, differing from it only in point of cheapness, costing but a little more than one-half the former.

The true place of this mode of treatment cannot be established until the experience of careful observers has been given us, years hence. I wish, therefore, to urge the profession to investigate the matter fairly, since time,

I am confident, will prove that Dr. Bergeon has been one of the greatest benefactors of the age.

DISCUSSION.

Following the reading of his paper, DR. CRANE laid before the society a letter from Dr. Bruen, of the Philadelphia Hospital, in which the writer states that the experience thus far obtained in that hospital in the use of Bergeon's treatment of consumption by rectal injections of carbonic acid gas impregnated with sulphuretted hydrogen, has been highly encouraging.

The clinical results indicate that the element of suppuration in pulmonary phthisis has been usually very positively influenced. The night sweats have been controlled in twenty-five cases, without an exception. The temperature has been modified, even in the most advanced cases, and in some instances in which the disease has been extensive it has been brought to a normal point. A peculiarity of the temperature charts seems to be an introduction of a subnormal temperature in the forenoon. The expectoration has been lessened in all instances, and in some has disappeared. A marked impression has been made for the better on the nervous symptoms which attend the disease, and a more cheerful and sanguine spirit has followed the therapeusis. One case of acute broncho-pneumonia, treated on this plan, has recovered, and will be discharged from the hospital. Another case of phthisis with pneumo-thorax, under treatment for five weeks, is so much improved that the patient insists on her discharge. The rales have disappeared from this patient's chest (the pneumo-thorax was local and confined to the lower zone of the right thorax), the opening into the lung has apparently closed, and retraction of the chest wall is going on. This patient had been ill for a year previous to admission to the Philadelphia Hospital; the pneumo-thorax was noted on admission. She has gained over nine pounds of flesh, expectoration has ceased, sweats abolished. The cough, however, continues as a modified symptom, since she is as yet merely a case of phthisis in stage of arrest, the bacilli of tuberculosis being still recognizable in the sputa.

Two cases of commencing phthisis with moist rales, dullness on percussion, harsh breathing, deficient expansion over right upper lobe, and tubercle bacilli in sputa, have recently been placed under the treatment. In these cases in one week the rales have disappeared and the patients express themselves as benefitted.

A solution of 5 grs. sulphide of sodium and 5 grs. chloride of sodium in 22 ounces of water has proven the most satisfactory substance to produce the sulphuretted hydrogen. A solution more strongly impregnated with sulphuretted hydrogen has not only not been advantageous, but seemed injurious, apparently inducing pallor, loss of appetite, and in some cases, irritation of the bowels. The gas should be introduced slowly; not less than half an hour seems requisite for each seance. From three to five quarts can be introduced in most cases, a gain in the tolerance of the necessary distension of the bowel being rapidly acquired. The absorption of the gas from the bowel is very rapid, and in half an hour the distension will entirely subside. It is sometimes introduced more rapidly, but the gain is in proportion to the time consumed in giving the gas. The carbonic acid can be pumped through the solution containing the sulphuretted hydrogen by the aid of a ball with valve attachment. Dr. Bruen has not obtained as yet satisfactory results from the examination of urine or breath, nor of the effect of the treatment upon the bacillus of tuberculosis.

Dr. R. H. Babcock said that his experience with this remedy is rather limited, but it seems there are several questions which should be considered in speaking on this subject: First, is the treatment safe? Second, is it applicable in all cases? Third, is it a cure? Practically the treatment is safe under the precautions which are laid down, but Dr. Osler, in some remarks before the Philadelphia County Medical Society, narrates a case in which the patient almost succumbed after the first treatment, the gas being charged with a mixture of bi-sulphide of carbon and sulphuret of hydrogen. We can also conceive of cases in which, on account of limited respiratory power, the rapid administration of the gas, by producing distension of the large bowel, would seriously embarrass the limited respiratory capacity remaining in the patient. It is also conceivable that an undue amount of distension in cases of tubercular ulceration of the bowel might be dangerous, possibly producing rupture and peritonitis; and it seemed to him this is a consideration which should require caution about administering this treatment in typhoid fever. In regard to its applicability in all cases, that has been partially answered in considering the previous question. It has been the experience of Geneva physicians, as reported by M. Wiss, and of De la Roche, of Lyons, that there are cases which cannot tolerate the treatment at all. One patient who had hæmorrhoids suffered from such colic that the treatment had to be abandoned. Wiss also mentions a case in which the treatment had to be abandoned in the course of a month, although superintended by Bergeon himself. He was asked to administer the treatment in a case in which the lungs were so extensively destroyed that it seemed unwise to administer it, though perhaps a small amount of gas might have ameliorated the symptoms; but had it been possible to abate or arrest the disease, the patient could certainly not have lived with such a small amount of lung left. Of course this consideration is one which renders the indiscriminate use of the treatment hazardous. There are cases in which the recovery or arrest of the disease has been so complete that the patients are practically well, and have been able to return to their avocations, However, if we are to estimate this thing by the disappearance of bacilli from the sputum we must say it is not a cure. No case is reported in which bacilli have entirely disappeared. As you knew, M. Cornil has instituted a number of experiments on animals with reference to this question, a solution of which all are looking for with much interest. French physicians are experimenting with a great many other substances.

He is now using the treatment upon two cases; one is bronchitic asthma, but the treatments are too few as yet to make any report. Another case is a lady aged 34, who has been suffering from phthisis for a year and a half, and who last fall received injections of phenic acid twice daily, without any improvement, who had been taking cabinet treatment for six weeks without improvement, in whom the right lung is extensively consolidated, with a large cavity; in this case the relief for the first week was certainly very surprising. The patient gained in strength and experienced a feeling of well-being, or, as she expressed it, a clearer feeling in her head, a feeling of brightness which she had not experienced in weeks. She certainly coughed less, and expectorated less at a time. Her stools had been clay-colored in spite of everything that could be done; at the end of a week they were of a natural color. The movement of the bowels was perfectly natural, her tongue also cleaned off in a remarkable manner at the end of five days. The treatments were continued twice a day, and the patient had to come into the city, so the improvement was perhaps not quite so great as if she had taken the treatment at home. The second week she did not do quite as well, which was attributed to the quality of the mineral water. At the end of two weeks she was improved in respect to all the symptoms which have been related this evening. The night sweats had ceased; her temperature was lower but not normal—it ranged from 99° to 100½°. Her tongue did not remain clean, and her appetite did not improve. She gained strength but there was a complication, namely, tuburcular laryngitis. M. Bergeon states decidedly that tuburcular laryngitis has been very greatly improved by this treatment. In the case just mentioned the laryngeal symptoms have improved, but whether entirely due to the treatment one cannot say, as she has had various sprays and one topical treatment to the larynx Her case is somewhat discouraging, owing to the extent of the disease, since it is not entirely confined to the right lung. She has complalned of no unpleasant symptoms since the first treatment, when she complained of a slight burning at the anus and a desire to evacuate the rectum. Once or twice she complained of a little colic. She is now taking the treatment at home and feels very much encouraged, but the outlook is not what it should be.

All the precautions have been taken in this case thus far, and it proves that the treatment does not give rapid improvement in all cases, and the fact cited by Wiss, that out of four cases treated in Geneva all died within three months, shows that it does not cure in all cases. However, it is a treatment which is so admirable and furnishes such good results in many cases, that all physicians are justified in trying it. Another thing which recommends it is, that after the patient becomes accustomed to the treatment it can be handed over to a trusty member of the family to administer. Compared with pneumatic differentiation it offers a much better chance for the improvement of the patient, although there are cases where pneumatic differentiation will do what this will not. But thus far this method gives better prospects of benefitting a number of cases than any method which has been placed before the profession.

DR. W. H. WEAVER: A case under treatment at the Chicago Policlinic, which is making favorable progress, is one in which there is no doubt of the extent of the tuberculosis in the left upper lobe. The expectoration was quite profuse and contained an abundance of bacilli. She has been failing for two years, but very rapidly for two months. She had fever, night sweats, prostration, wasting, loss of appetite, but had not taken any medicine of any sort. She came to the Policlinic dispensary for treatment; the first time the injection was only one pint of gas; her temperature that day was $101\frac{1}{2}$; the next day she returned and she had a quart injection and her temperature was then $102\frac{3}{5}$; the next day, the third injection, her temperature was normal and she had another quart. When she returned for the fourth injection her temperature was normal and she seemed better in every way; said her appetite had returned, the cough was less severe, expectoration diminished greatly, and the night sweats almost entirely relieved. At the fifth injection she had a slight temperature; she said she had had no more chills and fever for the last three days. He has given it in three other cases; one has some pelvic trouble and the physician who is treating her thinks that might be a counter-indication to the remedy, but she seems to be better in her general condition, has increased in flesh, and prefers to continue the treatment. Another case of incipient tuberculosis, where the upper left apex is affected more particularly, seems to be improved considerably. He has been having the treatment one week.

DR. L. L. MCARTHUR said that in starting in with this treatment of phthisis he saw the difficulties that were going to arise in the apparatus, and thinks he can make some suggestions that will prove valuable. The apparatus presented to-night is not portable, and if the patients are to be treated at home they would either require a separate apparatus for each patient or else one must run the risk of breaking the apparatus. In the first place he thought of a method of collecting the carbonic acid gas, by taking one of those portable laughing-gas cylinders, to be obtained at any dental depot, to a soda-water fountain and having it charged with carbonic acid gas. There can be stored from one to 500 gallons of gas, equally chemically pure with that recommended. This apparatus would simply require the turning of a stop-cock in the tube or opening to permit the gas to escape at any rate desired. If found desirable to continue the use of sulphuretted water, instead of mixing the two gases together first, it could be done by using a wash bottle and a delivery tube. He believes an equally efficient method is to introduce into this cylinder a solution of sulphide of potassium, which furnishes for every ounce of the sulphide of potassium about a gallon of sulphuretted hydrogen. The carbonic acid formed liberates the HS from the sulphide introduced. The strength according to Bergeon's method is about half of 1 per cent. or a little less. By a little calculation it will be seen that the carbonic acid formed by the combination of the carbonic dioxide and water would set free the hydrogen in combination with the sulphur in the definitely desired quantities, so that it would not be necessary to use the sulphuretted water. In that way the gas could be introduced into the measuring bag and then into the rectum. The cylinder would hold a sufficient amount of gas for many treatments, and as it is easily portable, it could be taken to the houses of patients and not require them to come to the office.

DR. R. TILLEY had had no experience whatever in this method of treatment, but would like to say a word with reference to the apparatus which would make the measurement of the gases used more definite. If a bottle of the necessary size was filled full of carbonic acid gas, and then another bottle with a tube attached full of water, the latter could be attached with the necessary tubes and elevated just the same as a siphon, and the water introduced into the bottle containing the carbonic acid gas, and passed through the sulphuretted hydrogen water with the required rapidity, and thus it would come much more regularly than could be done by means of this spasmodic pressure of the bulb. He would like to call attention to the fact that there are on sale in this city large cylinders containing absolutely pure carbonic acid gas, imported under the auspices of the Prussian government. This gas is condensed to the liquid state at the place where it flows from the earth at a great depth, from the natural channels, and it is only a little dearer than that which is manufactured here.

He would like to say a word about the marked indistinctness that has characterized some few of the reports. The way in which bisulphide of carbon and sulphuretted hydrogen were confused and interchanged was perplexing. Both these agents are exceedingly poisonous, and it is well to bear it in mind. He had some experience with the sulphuretted hydrogen some years ago in the laboratory, and it was his experience that the inhalation of a small quantity was attended almost regularly with a crop of boils. In any case, it would be better to have a more definite idea of the quantities of these poisonous agents than can be obtained by the information given us to-night.

DR. WALTER M. FITCH: For generating the gas we use a common Wolf bottle with three corks, one for a stop-cock for the acid, one for the delivery tube, and one for the safety valve. This last must not be pressed in too tightly. To make the gas we use about a heaping tablespoonful of bicarbonate of sodium, covering that with cold water about two inches deep. If we use hot water we make a suds that will foam up and run into the bag. We use the bicarbonate of sodium and sulphuric acid diluted one part in four. The reason for using the sulphuric acid in preference to any other is, that being absolutely non-volatile, none of it will pass over into the bag of carbonic acid gas. To make the gas, the generating bottle is connected with the bag, which is emptied and tightly rolled in order to remove all the air. The generator should also be emptied of air by turning in some of the acid before it is connected with the gas bag. The bags usually hold about one-half of what they are marked—a three-gallon bag will hold about six quarts, etc. If we should over-distend it the corks would be blown out of the generator. For injecting the gas the stop-cock is turned off and the bag is connected with the bubbling tube, which extends to the bottom of the bottle of mineral water, so that the gas coming from the bag may pass down into the water, and, rising up through it, be medicated by the sulphur vapor which it contains. It is injected by means of a hand bulb. In using the bulb it is important to remember that, since it is intended to be used only with water, the action of the valves is not perfect with gas, and it is therefore necessary that the bulb be held vertically, so that the weight of the valves may assist in closing them. In using it in this way we secure a perfect action, which draws the gas into the bottom of the bottle and delivers it through the tube. The bisulphide of carbon has been mentioned several times. In order to use this it is necessary that the gas be washed, for fear it may contain impurities, for none of the chemicals we get are perfectly pure, and, as they are most likely to contain vapors of hydrocloric acid, which are easily dissolved in water, the gas is bubbled through pure water to purify it. Between the Wolf bottle and the bulb is inserted an ordinary chemical drying tube, a glass tube three-fourths of an inch in diameter, which is drawn down at one end small enough to have the rubber tube slipped over it. Into the other end is fitted a cork with a glass tube through it; in the drying tube is placed a tampon of cotton, and the requisite amount, usually one drachm, of bisulphide of carbon is placed on the cotton. In passing the gas through this cotton it becomes very strongly impregnated with the vapor and is injected in that manner. A very simple way of measuring the gas, when using a small apparatus of this kind, is with the bulb. The bulb will deliver at each compression just so much gas; in order to measure how much is delivered, we simply take a measured bottle, invert it over the water, with the water filling it, then introduce the rectal tube into the bottle and remove the water from it by displacement, the number of compressions being counted. The bulbs vary in size. Of six now in use, two count fifteen compressions to the pint; three count twenty compressions to the pint, and one counts twenty-five. Thus, with each apparatus the bulb must be measured, and knowing its capacity, the gas can be prescribed in as definite a way as any medicine measured out of a bottle.

THE PRESIDENT said his experience with this method, extending over less than a month, comprises the observation of one case that impressed him strongly. This was a young man suffering from the last stages of quick consumption, who had been in the care of Dr. H. A. Johnson. He was very feeble, much emaciated, had badly swollen feet and albuminous urine. His temperature ranged from 101 to 103 each afternoon, and he had profuse night sweats. In addition to these usual symptoms he suffered to an extraordinary degree from laryngeal tuberculosis. He had been unable for many weeks to lie down with comfort, and according to his own statement, had not slept fifteen minutes in three months. Cough was constant and expectoration profuse. About a month before Doctor Johnson had given him three or four weeks to live. Gaseous enemata, employing the artificial solution advised by Doctor Bruen, was used twice a day. Improvement began on the second day, and by the fourth day was pronounced. The cough was less distressing and the sweats and expectoration had diminished; the patient was able to lie down and sleep for two hours continuously. He was, however, so evidently near dissolution that it was deemed inadvisable to continue a tentative treatment, and stopped it on the fourth day. Two days later he sent an urgent request for the resumption of the treatment, saying that his former symptoms were returning

However, he declined to use the injections further, and six days after their discontinuance the patient died, nearly two weeks after his physician had expected his demise.

It should be remembered that these injections seem to arrest not the tuberculosis proper, but the sepsis which is secondary thereto, and that there are numerous other septic conditions in which benefit may fairly be expected, such as surgical sepsis, dipththeria, puerperal fever, etc. The apparatus furnished by the stores is unnecessarily expensive and inconvenient for a visiting practice. He uses an improvised apparatus consisting of a gas bag, double perforated cork, and ordinary large wide mouthed bottle—the whole costing $2.50 instead of $10. The entire outfit can be readily improvised at a little cost and carried in a small handbag.

His limited experience gives him the impression that the artificial solution of the sulphide of potassium is preferable to either Ypsilanti or Blue Lick water, because the amount of sulphuretted hydrogen in these waters as we obtain them at the drug store appears to be extremely variable, while in the artificial solution it can be maintained at a given percentage, increased or diminished as desired, yet care should be taken to make a weak solution about such as is recommended by Doctor Bruen, since a too-concentrated solution of the poison may produce very unpleasant symptoms. This method can be employed by every practitioner as well as by the most expert specialist. If it will accomplish nothing more than the relief of the distressing cough, expectoration, fever and sweats of advanced consumption, it will at least do what can be effected by no other therapeutic measure with which he is acquainted; hence it is to be hoped that it will be submitted to a general test by the profession, that we may quickly ascertain exactly its merits and demerits.

DR. C. M. FITCH: The remedies that have been ordered for consumption in the past are about as numerous as the sure-cures that have been offered for cancer and hydrophobia, and have commanded about as much attention from the profession. But this method of Bergeon's comes to us under very different auspices; it comes with a letter of introduction that demands from us respectful attention. J. Henry Bennett indorses Bergeon as a man worthy of credit; and if Bergeon's statements be true they are of the deepest interest to us all. It is in Bergeon's favor also that he not only asks us to believe something, but he gives us a reason for so believing —a plausible explanation of what he wishes us to receive. He assumes that septicæmia, arising either from the absorption of pus, or the morbid product of the bacilli, is the cause of the formidable symptoms of phthisis, and that if we can antagonize this septicæmia, we can undo day by day the deadly work of the bacilli, and shall gain time in which the lesions that have arisen from phthisis may be overcome.

The first case in which he had occasion to test this method of Bergeon was one of extreme anxiety to him. The patient, a young lady 20 years of age, had previously had exceptionally good health, was well nourished, with a muscular development that seemed to defy fatigue. She returned in July last from a visit to Colorado with a cough which was charged to the dryness of the climate and the inhalation of the alkaline dust, which causes a catharrhal irritation. This seemed a plausible explanation, and as the cough was not urgent, it received but little attention until, in November, she took a cold which resulted in chills for three or four successive days, and seemed to prostrate her greatly. He then examined her chest, and, to his astonishment, found extensive and marked consolidation at the apex of the left lung; there was also irritation throughout the remainder of the lung and some moist rales. Several of our best physicians tendered their aid, but the case failed to improve. The patient lost flesh rapidly, her cough became fearfully oppressive and her temperature never fell to normal, rarely below 100°, and most of the time 101° and rising to 103°. She had daily chills and profuse night sweats, and the case presented all the features of acute phthisis. At the last examination, made by Doctor Belfield and Doctor Ochsner, both thought they detected bacilli in the sputum, although previous examinations had failed to show them.

Late in January his attention was called to an article in the *Medical Record*, giving a notice of Bennett's paper in the *British Medical Journal*, which contained an account of Bergeon's method. After considerable trouble he obtained a copy of the paper which contained this article, and went to Sargent's to have an apparatus made, and there learned that they had just made one for Dr. Crane, who kindly allowed him to appropriate it and ordered another for himself. Knowing nothing at that time of what he might expect in such a case from our different mineral waters, and knowing that Dr. Bergeon recommended bi-sulphide of carbon as the best substitute, he commenced treatment with the bi-sulphide, and the effect was surprising. The treatment was commenced seven weeks ago to-day; during the week previous the temperature had at no time fallen to normal; the lowest point was 100°, and once it rose to 105½°, and night sweats and chills were constant. The first time the injection was tried very cautiously, only a moderate amount of gas being used. The effect was not specially apparent; the second day the temperature still rose to 103°. That night the gas was used much more freely, and with results that to us were somewhat alarming. The next morning the temperature fell to 95°, and it did not rise above 100° during the day; the cough was almost suppressed, although it had previously been very distressing. The next day we did not use the gas quite so freely, but the day following the lung was almost entirely occluded; the mucus seemed to become viscid, and the air was almost shut out from the lung. There was so little elimination of the bi-sulphide through the lung that we soon began to have marked symptoms of bi-sulphide poisoning, although in many respects she appeared better; the cough was almost gone, and there were no chills. The treatment was discontinued for about ten days, and then resumed with the Ypsilanti mineral water. From that time there has been a satisfactory improvement, and she is better than she has been at any time since her sickness began; her appetite and strength are improved, and she is evidently beginning to gain flesh; her cough is greatly lessened, and the chills have altogether disappeared. Her night sweats have been absent for six weeks; although during the last warm weather she perspired a little for three or four nights, there was nothing in the nature of colliquative sweating, and she seems now in a fair way to recover.

The second case in which he tried this remedy seemed about as hopeless as well could be; the patient was confined to her bed, had been suffering from severe hæmorrhage, chills, night sweats, and great prostration. There was marked dullness at the apex of the right lung, and the physician who was treating her recognized softening. The husband, whose family were patients of his, after the physician told him that nothing more could be done, came to him. He told him that he did not know that he could do anything more; the only thing he could suggest would be this new treatment which he had been experimenting with, and which might be of some value. He was anxious to have something tried, and the case was undertaken. At the end of four days this patient's chiils, which had existed then about a month, ceased entirely, and have not since recurred. On the ninth or tenth day the sweats disappeared; her cough was relieved from the first, no opiate could have controlled it more completely. The expectoration was also lessened, her appetite has improved, and she is gaining in strength. In all respects he thinks she has gained as much in the time she has been under treatment as he ever saw a patient gain under any treatment in the same time.

Another case was a young man of scrofulous habit, who has a stiff knee from scrofulous disease of the joint. He has been under treatment about three weeks. There was marked consolidation in his case, also, at the apex of the right lung, and every indication of phthisis. He has improved materially; his cough is much lessened and he is gaining in strength. The indigestion and flatulence which had previously troubled him very much, has disappeared under the use of the gas; there has also been a great improvement in his appetite.

A fourth case was a young woman of twenty, who came under treatment some two or three weeks ago.

The case was chronic, was not urgent in character, at the same time he thought there was little doubt of its being a true case of phthisis. She gained rapidly from the first, and at the end of ten days her menses, which had been absent since December, returned, and her cough was so nearly cured that she considered herself well, and without consulting her physician discontinued the treatment.

Another case, that of laboring man, large and muscular, who has been working where there is a great deal of dust, probably from mechanical irritation, had contracted trouble with his lungs. He had repeated hæmorrhages, which caused great loss of strength; there was marked though limited dullness at the apex of the right lung, but the chief feature of the case was the marked loss of strength, altogether too great to be accounted for by the lesion in the lung. There seemed to be some torpidity of the liver, and a little proto-iodide of mercury was given. His strength, either rom the gas or stirring up the liver, has very greatly improved.

The other cases he had seen have not been under treatment long enough to justify a report. It seems that we have a remedy that is worthy further trial. Whether it will fulfil the promise of its youth we have yet to see. He had no doubt this remedy will relieve spasmodic asthma, and there are other conditions that it would relieve. In puerperal fever he should try it, using the bi-sulphide of carbon, but using it cautiously. He also thinks it would be of value in malignant diphtheria and typhoid fever.

